# Biocompatibility assessment of single-walled carbon nanotubes using *Saccharomyces cerevisiae* as a model organism

**DOI:** 10.1186/s12951-018-0370-1

**Published:** 2018-04-25

**Authors:** Song Zhu, Fei Luo, Jian Li, Bin Zhu, Gao-Xue Wang

**Affiliations:** College of Animal Science and Technology, Northwest A&F University, Xinong Road 22nd, Yangling, 712100 Shaanxi China

**Keywords:** Carbon nanotubes, Yeast, Uptake, Oxidative stress, Apoptosis

## Abstract

**Background:**

Single-walled carbon nanotubes (SWCNTs) have many potential applications in various fields. Especially, the unique physicochemical properties make them as the prime candidates for applications in biomedical fields. However, biocompatibility of SWCNTs has been a major concern for their applications. In the study, biocompatibility of oxidized SWCNTs (O-SWCNTs) was assessed using *Saccharomyces cerevisiae* (*S. cerevisiae*) as a model organism.

**Results:**

Cell proliferation and viability were significantly changed after exposure to O-SWCNTs (188.2 and 376.4 mg/L) for 24 h. O-SWCNTs were internalized in cells and distributed in cytoplasm, vesicles, lysosomes and cell nucleus. The average O-SWCNTs contents in *S. cerevisiae* were ranged from 0.18 to 4.82 mg/g during the exposure from 0 to 24 h, and the maximum content was reached at 18 h after exposure. Both penetration and endocytosis were involved in the internalization of O-SWCNTs in *S. cerevisiae*, and endocytosis was the main pathway. Cellular structures and morphology were changed after exposure to O-SWCNTs, such as undulating appearance at the membrane, shrinking of the cytosol, increased numbers of lipid droplets and disruption of vacuoles. ROS and antioxidant enzymes activities were observably changed following exposure. For the treatment at 376.4 mg/L, 20.8% of the total cells was undergone apoptosis. Decrease of mitochondrial transmembrane potential and leakage of cytochrome c from mitochondria were observed after exposure. Moreover, expression levels of apoptosis-related genes were significantly increased.

**Conclusions:**

O-SWCNTs can internalize in *S. cerevisiae* cells via direct penetration and endocytosis, and distribute in cytoplasm, vesicles, lysosomes and cell nucleus. Besides, O-SWCNTs (188.2 and 376.4 mg/L) can induce apoptosis in *S. cerevisiae* cells, and oxidative stress is involved in activation of the mitochondria-dependent apoptotic pathway.
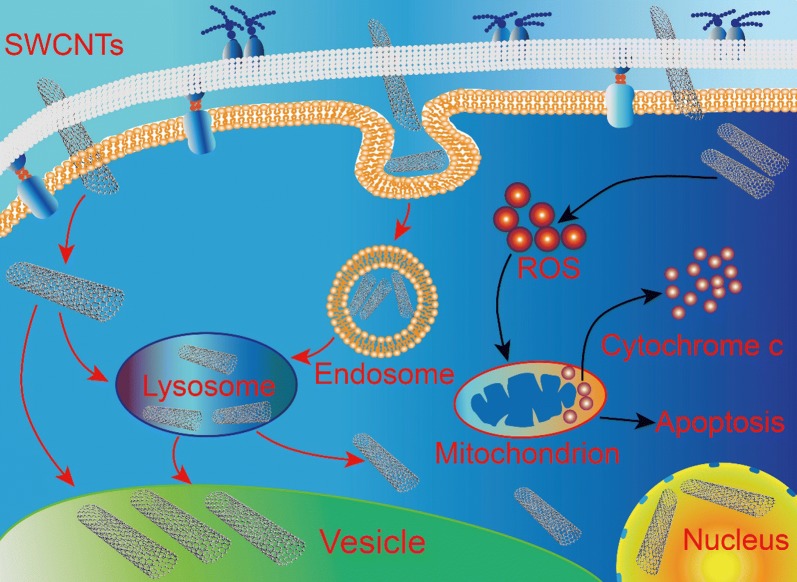

## Background

Single-walled carbon nanotubes (SWCNTs) are important nanomaterials that have potential to be used for a variety of applications in many fields [10]. Especially, they have the potential to revolutionize biomedical research. Merging of SWCNTs with medicine presents an unprecedented opportunity for developing novel materials that can improve treatment and diagnosis of diseases. Several highly promising applications have been reported in some studies, such as drug delivery, gene therapy, growth substrates, tissue scaffolds and biological imaging [2, 3, 17]. When considering their biomedical applications, SWCNTs are expected to have an intrinsically low systemic toxicity [18]. Therefore, the biological, environmental and safe profiles of SWCNTs need to be thoroughly characterized and understood before their broadly used.

A growing number of studies have assessed the potential toxicity of SWCNTs to a number of cell types and organisms [12]. Some studies reported that SWCNTs can internalize in cells and induce cell apoptosis/necrosis. Besides, one of the most common SWCNTs mechanisms that lead to cytotoxicity is related to oxidative stress that can cause mitochondrial impairment and apoptosis [9, 30, 36]. The uptake pathways and intracellular distribution of SWCNTs have also been investigated in previous studies. Data so far supported that endocytosis is the major uptake pathway of SWCNTs in mammalian cells, and SWCNTs are distributed in cytoplasm, lysosomes, mitochondria and cell nucleus [33]. The complexity of SWCNTs toxicity is attributed to various parameters, such as purity, length, agglomeration and surface functionalization of SWCNTs, especially, different types of cells and organisms were used in the toxicity studies [33, 41]. Owing to their lightweight characteristics, SWCNTs are ubiquitously distributed in the environment, including air, water and soil. Various kinds of organisms that active in the environment are at risk of SWCNTs. Therefore, representative organisms are necessary to systematic study the uptake, distribution, accumulation and biocompatibility of SWCNTs.

*Saccharomyces cerevisiae* (*S. cerevisiae*) is one of the most intensively used unicellular eukaryotic model organisms in molecular and cell biology studies. Its cellular structure and functional organization share much similarity with cells of plants and animals, furthermore, it also has a short generation time and can be easily cultured like bacteria. Therefore, toxicity studies using *S. cerevisiae* as a model organism can provide clues to understand nanotoxicity in higher organisms and bacteria [14, 20, 34]. *S. cerevisiae* is a widely used model organism for the study of oxidative stress and apoptosis [7], and its genome was sequenced [15]. Thus lots of data and genes are available for mechanistic studies. It has been widely used as a model organism in the toxicological studies of nanomaterials, such as TiO_2_, ZnO, CuO, Al_2_O_3_, Mn_2_O_3_, CeO_2_ and SiO_2_ nanoparticles [14, 20, 34]. In addition, we have investigated the effects of multi-walled carbon nanotubes (MWCNTs), graphene oxide (GO) and α-Fe_2_O_3_ nanoparticles on *S. cerevisiae* [45–47]. Results showed that *S. cerevisiae* is a suitable model organism for toxicological studies of nanomaterials.

In the study, biocompatibility of oxidized SWCNTs (O-SWCNTs) was assessed using *S. cerevisiae* as a model organism. Based on previous studies, we hypothesized that (1) O-SWCNTs would be internalized and well distributed in *S. cerevisiae*; (2) both penetration and endocytosis would involve in the internalization of O-SWCNTs in *S. cerevisiae*; (3) O-SWCNTs would induce apoptosis in *S. cerevisiae* cells; and (4) oxidative stress would involve in activation of the mitochondria-dependent apoptotic pathway. The study contributes to a better understanding of the O-SWCNTs biocompatibility, and lays foundation for their applications.

## Methods

### Characterization of O-SWCNTs

SWCNTs (Chengdu Organic Chemicals Co., Ltd., China) were purified and oxidized according to our previous study [44]. O-SWCNTs (600 mg) were weighed on aluminum foil and dispersed in 1 L YPD medium (1% yeast extract, 2% peptone and 2% glucose). The resulting suspension was sonicated for 2 h at 100 W with a 50% on/off cycle. Following the sonication, the suspension was centrifuged at 12,000*g* for 2 h. The supernatant was collected, and concentration of O-SWCNTs was determined by Raman spectrophotometer. Raman spectrophotometer is a useful tool to quantify CNTs due to the normalised G-band areas show linear concentration behaviour in accordance with Beer’s law [4, 16, 39]. For the quantitative analysis, a standard curve between the concentrations of O-SWCNTs and the normalised G-band areas was established. Then, the supernatant was pipetted into a square quartz groove (side length: 5 mm, height: 1 mm) and dried in vacuum at 80 °C. Contents of O-SWCNTs were measured using the Raman spectrophotometer with an excitation wavelength of 785 nm, and calculated by the standard curve. The supernatant was diluted to create suspensions with a series of concentrations by double dilution method. All suspensions were sterilized and sonicated again before used. For SWCNTs characterization, both SEM (Hitachi S-4800, Japan) and TEM (JEM1200EX, Japan) were used to observe the shape of O-SWCNTs. The purity, chemical states and elemental compositions of pristine SWCNTs (P-SWCNTs) and O-SWCNTs were analyzed using Raman spectrophotometer (Longjumeau Cedex, France) and XPS (PHI-5600, Russia).

### Cell proliferation and viability

*Saccharomyces cerevisiae* was cultivated in O-SWCNTs suspensions with constant shaking (160 rpm) at 30 °C, and the inoculation quantity was approximately 1 × 10^5^ cells/mL. For proliferation assays, cells were counted at 0, 3, 6, 9, 12, 15, 18, 21 and 24 h with a hematocytometer under an optical microscope (Olympus Optical Co., Ltd., Tokyo, Japan). To check the effect of O-SWCNTs on cell viability, cells were collected following exposure for 24 h and stained with 1 mg/mL Trypan Blue (Sigma, USA) for 3–5 min. The stained cells and total cells were counted under the microscope, and mortality rate was calculated as a ratio between stained cells and total cells. All measurements were carried out eight times.

### Internalization, distribution and uptake kinetics of O-SWCNTs in *S. cerevisiae*

Internalization and distribution of O-SWCNTs in *S. cerevisiae* were observed using the TEM according to Bayat et al. [6]. In order to measure the uptake kinetics, contents of O-SWCNTs in *S. cerevisiae* were quantitatively assessed after exposure for 0, 3, 6, 9, 12, 15, 18, 21 and 24 h. Dry weight of *S. cerevisiae* cells was measured before the quantitative assessment. Briefly, cells treated without O-SWCNTs were collected and counted under the microscope. Then, the cells were dried using a freeze dryer (FD5-3, GOLD-SIM) and weighted using a balance (Sartorius Stedim Biotech GmbH, Germany). After that, the average weight of a single cell was calculated. The O-SWCNTs contents were measured based on the dry weight of *S. cerevisiae*, reflecting the total cell burden across the exposure from 0 to 24 h. For the measurement, cells were collected using density gradient centrifugation [47] at each time point, and then counted under the microscope. The cells were pipetted into a square quartz groove (side length: 5 mm; height: 1 mm), and then dried in vacuum at 80 °C. Contents of O-SWCNTs were measured using the Raman spectrophotometer with an excitation wavelength of 785 nm, and calculated by the standard curve. All measurements were carried out three times.

### Uptake mechanism

To elucidate whether O-SWCNTs enter into *S. cerevisiae* via endocytosis, cells were exposure to O-SWCNTs suspension in the presence of 5% (v/v) ethanol and at 4 °C, respectively. O-SWCNTs in *S. cerevisiae* were quantitatively measured using Raman spectrophotometer as described above. Moreover, expression levels of endocytosis-related genes (END3, END6, Sla2 and Rsp5) were measured by Real-Time PCR using ribosomal 18S RNA (18S rRNA) as internal standard. Primers for the genes and cycling conditions were designed as previous studies [47]. Relative expression was obtained by using the 2^−∆∆Ct^ [40] method and normalized to the expression of the internal standard gene 18S rRNA in the same sample. All measurements were carried out three times.

### Effects of O-SWCNTs on cellular structure

After exposure for 24 h, cells were collected using density gradient centrifugation and washed with PBS (pH = 7.1). The cells were fixed with 2.5% glutaraldehyde in PBS at 4 °C for 24 h and then adhered onto a piece of glass using polylysine. Afterwards, cells were dehydrated through a graded ethanol series (30–90%, 2 × 100%; 15–20 min each) and replaced using isoamyl acetate. The samples were dried overnight and coated with a thin layer of gold, and observed using the SEM. Moreover, effects of O-SWCNTs on cellular structure were also checked using TEM according to Bayat et al. [6].

### ROS and antioxidant enzymes activities

After exposure for 24 h, approximately 1 × 10^8^ cells were collected. The cells were mixed with glass beads (0.3–0.4 mm) and thoroughly ruptured by vigorous vortexing for 5–10 min. After vortexing, the homogenates were centrifuged (12,000 rpm, 4 °C) for 10 min. The supernatants were collected for ROS and antioxidant enzymes (SOD, CAT and GPx) activities measurements. Total protein, ROS, SOD, CAT and GPx activities were measured using kits (Nanjing Jiancheng Bioengineering Institute, Nanjing, China) according to manufacturer’s instructions, and detected by a microplate reader (Multiskan MK3, Thermo Labsystems Co., Beverly, MA). All measurements were carried out three times.

### Apoptosis assay

After exposure for 24 h, approximately 2 × 10^5^ cells were collected from each treatment and stained with annexin-V-FITC (5 μL) and PI (5 μL) following the manufacturer’s instruction. Following the staining, flow cytometry (Beckman Coulter Inc., United States) analysis was immediately conducted. FITC fluorescence (FL1) and PI fluorescence (FL2) of each cell were quantitated using the Cell Quest Pro^®^ software (BD, Germany).

### Mitochondrial transmembrane potential

Mitochondrial transmembrane potential (MTP, ∆ψ_m_) was detected using JC-1 (Beyotime Biotech, Nantong, China) as described in previous studies [8, 35]. Briefly, cells were collected following exposure for 24 h. The cells were incubated with JC-1 following the manufacturer’s instruction and analyzed using a microplate reader (Multiskan MK3, Thermo Labsystems Co., Beverly, MA). Ratios between red and green fluorescent were calculated, and results were shown as percentage between the ratios of treatments and that of the control. All measurements were carried out three times.

### Cytochrome c leakage from mitochondria

After exposure for 24 h, approximately 1 × 10^6^ cells were collected from each treatment. The cells were ruptured as described above, and supernatants were collected. A small amount of Na_2_S_2_O_3_ was added into the supernatants in order to keep cytochrome c in the reduction state. Absorbance of cytochrome c was measured at 540 nm using a UV–visible spectrophotometer (UV-2200, Shimadzu, Japan). All measurements were carried out three times.

### Expression of apoptosis-related genes

Expression levels of apoptosis-related genes (SOD, Yca1, Nma111 and Nuc1) were measured by Real-Time PCR as described above. All measurements were carried out three times.

### Statistical analysis

Data were expressed as mean ± standard deviation (SD) and analyzed using SPSS Version 11.0 software package (SPSS Inc., Chicago, IL). Differences between the controls and treatments were analyzed using one-way ANOVA followed by Tukey’s test, where *p *< 0.05 was considered significant.

## Results and discussion

### Characterization of SWCNTs

**Fig. 1:**
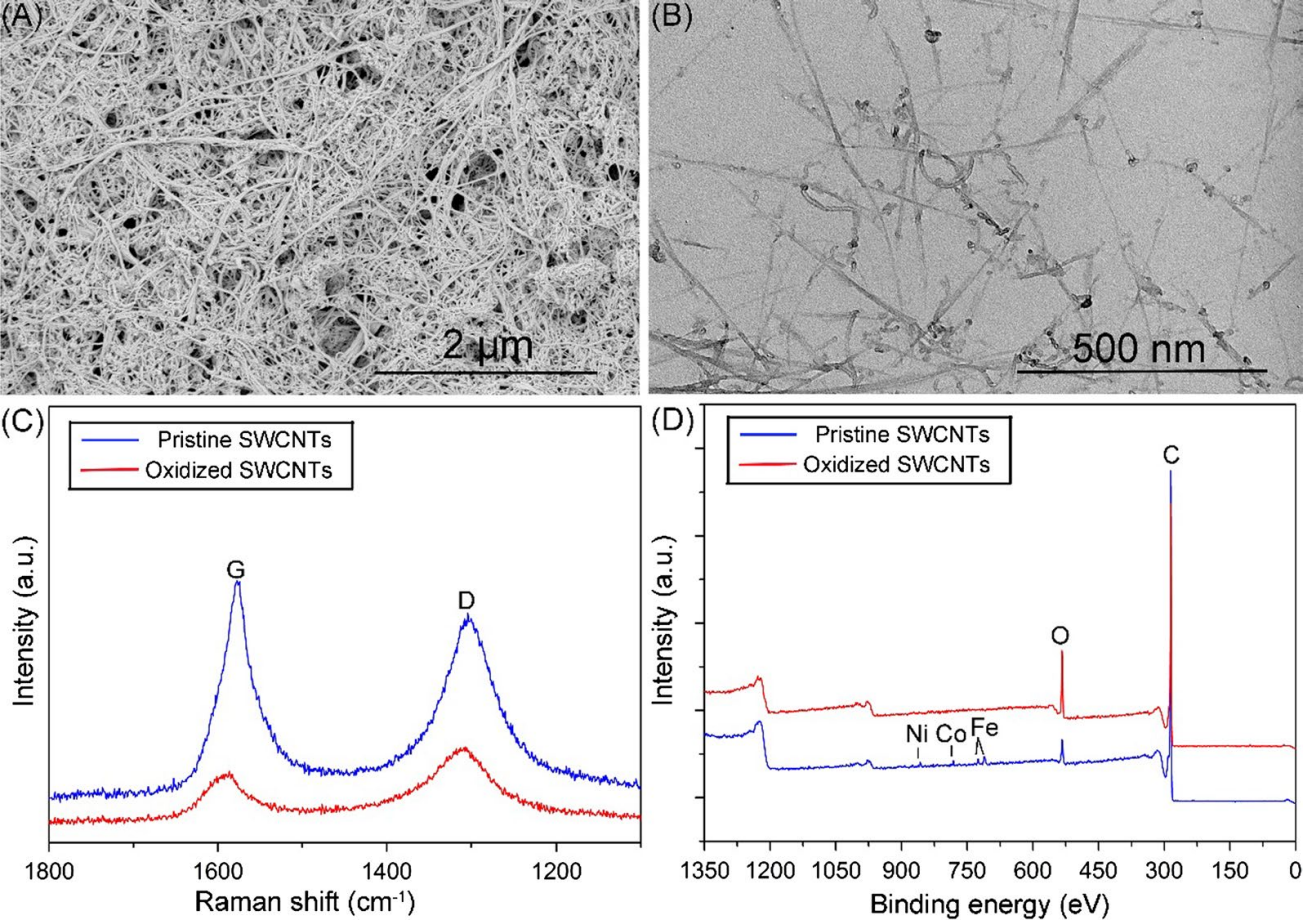
Characterization of SWCNTs. SEM (**a**) and TEM (**b**) images of O-SWCNTs. Raman (**c**) and XPS (**d**) spectra of P-SWCNTs and O-SWCNTs

The complexity of SWCNTs toxicity is attributed to various parameters, such as concentration, purity, length and surface functionalization [33]. Therefore, these parameters should be well characterized before the toxicity assessment. In the study, O-SWCNTs suspension was sonicated and centrifuged to acquire well-dispersed supernatant. Concentration of O-SWCNTs in the supernatant was 376.4 mg/L. The supernatant was diluted to create suspensions (23.5, 47.1, 94.1 and 188.2 mg/L) by double dilution method. As shown in SEM (Fig. 1a) and TEM (Fig. 1b) images, O-SWCNTs were fibrous with varying lengths. According to statistical analysis of TEM images, the average length of O-SWCNTs was 263 nm, and shorter than the average length of P-SWCNTs (3.16 μm). As shown in Fig. 1c, typical G (around 1580 cm^−1^) and D band (around 1303 cm^−1^) of CNTs [11] were identified from the raman spectra of P- and O-SWCNTs. D/G band intensity ratio (I_D_/I_G_) can be used to monitor the defects of CNTs as it is increased with the defects increasing [28, 31]. The I_D_/I_G_ ratios of P-SWCNTs and O-SWCNTs were 0.82 and 1.32, indicating that defects of SWCNTs were increased following oxidation. Figure 1d shows XPS spectra of P- and O-SWCNTs, indicating SWCNTs surface was consisted of carbon (284 eV) and oxygen (532 eV). Surface oxygen contents for P- and O-SWCNTs were 3.32 and 14.6 atomic %, respectively, suggesting the oxidation was sufficient. There are small amounts of iron [711 (Fe 2p_1/2_) and 725 (Fe 2p_3/2_) eV], cobalt (782 eV) and nickel (850 eV) in P-SWCNTs which not found in O-SWCNTs, indicating that O-SWCNTs were free from impurities following oxidation. All the results showed that the oxidation of SWCNTs was sufficient and the impurities were removed.

### Effects of O-SWCNTs on cell proliferation and viability

**Fig. 2:**
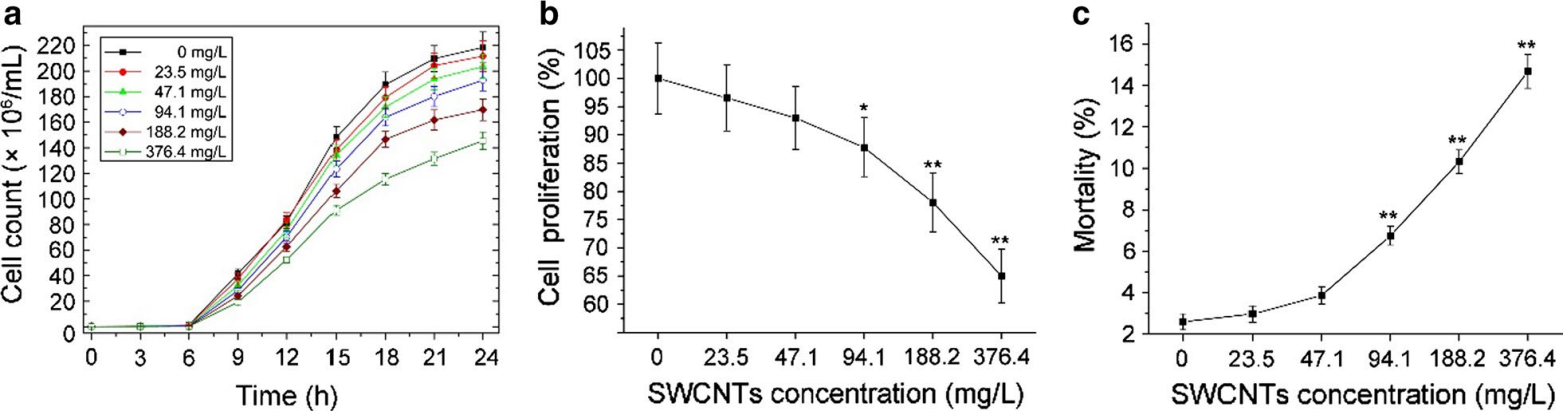
**a** Growth curves of *S. cerevisiae* exposed to 0–376.4 mg/L O-SWCNTs suspensions. Effects of O-SWCNTs on cell proliferation (**b**) and viability (**c**) after exposure for 24 h. Values are presented as mean ± SD. Values that are significantly different from the control are indicated by asterisks (one-way ANOVA, *p < 0.05; **p < 0.01)

After exposure to O-SWCNTs suspensions, cell proliferation showed a dose-dependent inhibition (Fig. 2a), and was significantly inhibited (*p *< 0.01) at 188.2 and 376.4 mg/L (Fig. 2b). The cells proliferation at 376.4 mg/L was 64.9% compared with the control after exposure for 24 h. Mortality was notably increased (*p *< 0.01) at 94.1–376.4 mg/L, and was 14.7% after exposure to 376.4 mg/L for 24 h (Fig. 2c). Effects of MWCNTs on *S. cerevisiae* have been investigated in our previous study [47]. Proliferation and mortality after exposure to 400 mg/L MWCNTs were 72.2% (compared with the control) and 6.1%, respectively. Therefore, these data indicated that SWCNTs possess a higher toxicity file than MWCNTs.

### Internalization, distribution and uptake kinetics of O-SWCNTs in *S. cerevisiae*

**Fig. 3:**
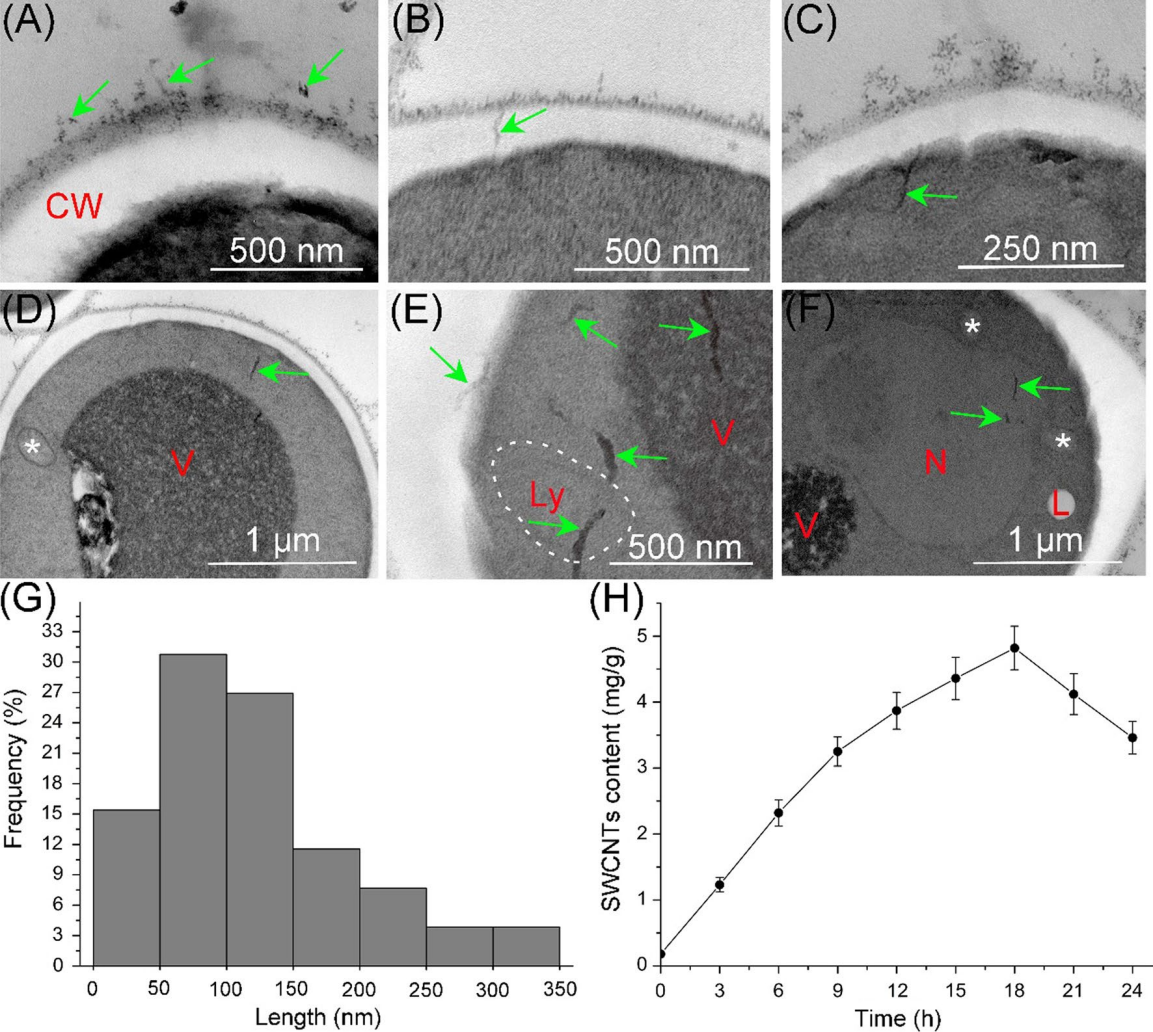
Internalization, distribution and uptake kinetics of O-SWCNTs (green arrows) in *S. cerevisiae*. O-SWCNTs were firstly adsorbed onto the cell wall surface (**a**) and then penetrated across the cell wall (**b**) and membrane (**c**). O-SWCNTs entered into the cells and distributed in cytoplasm (**d**), vesicles and lysosomes (**e**), and cell nucleus (**f**). **g** Length distribution of O-SWCNTs in *S. cerevisiae* cells. **h** Uptake kinetics of O-SWCNTs in *S. cerevisiae* cells. Values are presented as mean ± SD. *CW* cell wall, *V* vesicle, *Ly* lysosome, *N* nucleus, *L* lipid droplet, *mitochondrion

Several studies about the distribution of SWCNTs have been reported and showed that SWCNTs distributed in the cytoplasm, lysosomes, mitochondria, endosome-like vesicles and cell nucleus [33, 37]. The internalization, distribution and uptake kinetics of O-SWCNTs in *S. cerevisiae* were checked in the study. As shown in Fig. 3a, O-SWCNTs were firstly adsorbed onto the cell wall surface and then penetrated across the cell wall (Fig. 3b) and membrane (Fig. 3c). Subsequently, O-SWCNTs entered into the cells and distributed in cytoplasm (Fig. 3d, e), vesicles (Fig. 3e), lysosomes (Fig. 3e) and cell nucleus (Fig. 3f). These results were similar to that reported by Porter et al. [37], who demonstrated that SWCNTs distributed in cytoplasm, lysosomes, endosome-like vesicles and cell nucleus of human monocyte-derived macrophages [37]. We investigated the distribution of MWCNTs in *S. cerevisiae*, and showed that MWCNTs were distributed in the perinuclear region without entering into cell nucleus [47]. The difference may be due to that SWCNTs have a smaller diameter and stronger penetrability than MWCNTs.

As shown in Fig. 3g, the average length of SWCNTs in cells was around 110 nm, which was shorter than the SWCNTs (263 nm). The result indicated that shorter SWCNTs are more easily enter into cells. The uptake kinetics of O-SWCNTs in *S. cerevisiae* was shown in Fig. 3h. The average O-SWCNTs contents were ranged from 0.18 to 4.82 mg/g, and the maximum content was reached at 18 h after exposure. A decrease was observed from 18 to 24 h probably due to the discharge of O-SWCNTs. Uptake kinetics of MWCNTs in *S. cerevisiae* was investigated in previous study. Result showed that the maximum MWCNTs content was reached at 3 h after exposure [47], indicating that SWCNTs presented a slower uptake compared with MWCNTs.

### Uptake mechanism

**Fig. 4:**
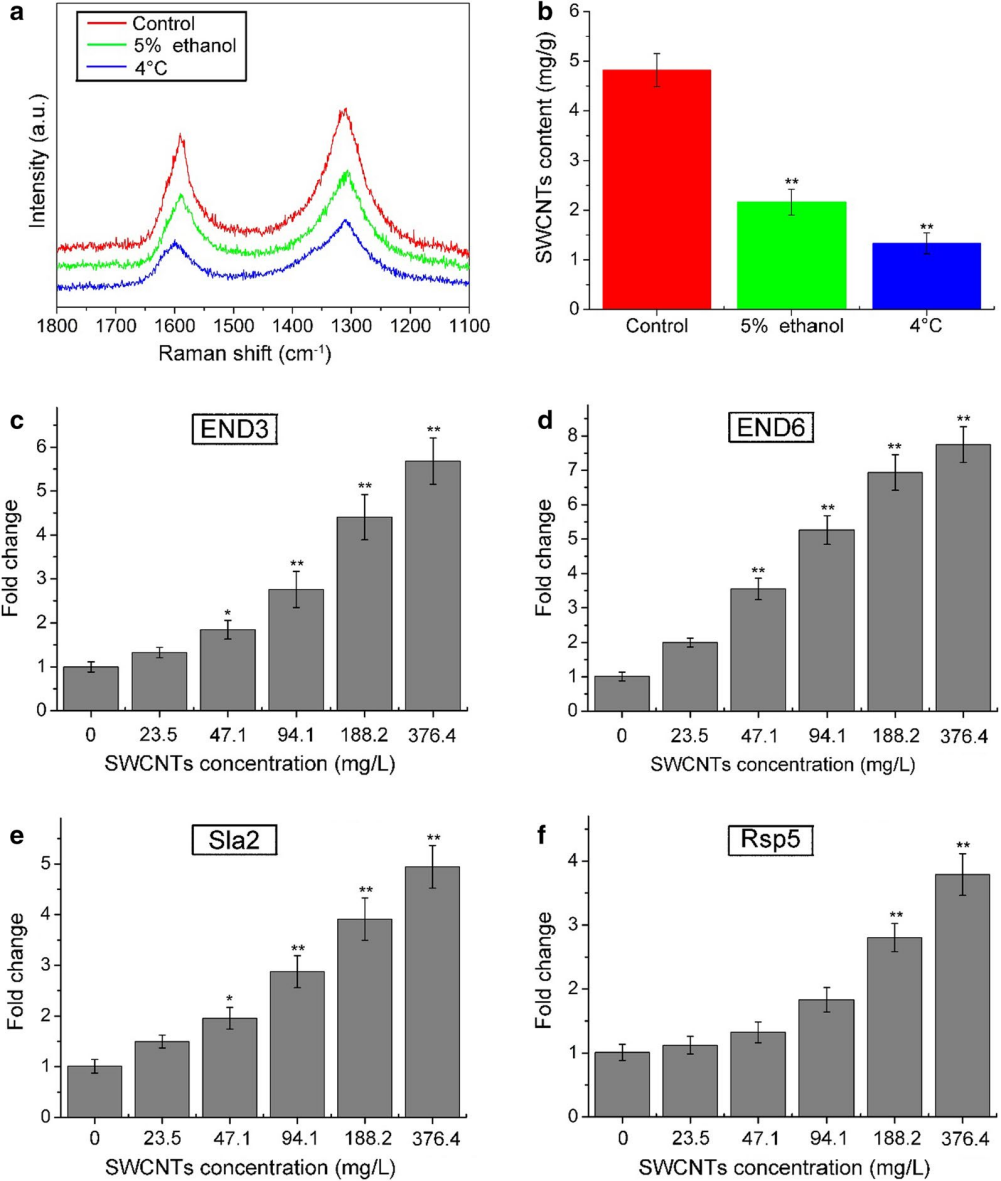
Internalization of O-SWCNTs in *S. cerevisiae* cells via endocytosis. Raman spectra (**a**) and contents (**b**) of O-SWCNTs uptake by *S. cerevisiae* cells under different treatment (control, 5% ethanol and 4 °C). Expression levels of endocytosis-related genes [END3 (**c**), END6 (**d**), Sla2 (**e**) and Rsp5 (**f**)] after exposure to 0–376.4 mg/L O-SWCNTs suspensions. Values are presented as mean ± SD. Values that are significantly different from the control are indicated by asterisks (one-way ANOVA, *p < 0.05; **p < 0.01)

Several findings suggested that endocytosis is the major uptake pathway of SWCNTs in mammalian cells [19, 33]. To investigate whether endocytosis occurs for the internalized O-SWCNTs in *S. cerevisiae* cells, we studied the effect of endocytosis inhibiting conditions (5% ethanol [23] and 4 °C [29]) on cellular uptake. As shown in Fig. 4a, b, the contents of O-SWCNTs in cells were significantly decreased after incubation with 5% ethanol (2.16 mg/g) and at 4 °C (1.33 mg/g) compared with the control (4.82 mg/g). Contents of O-SWCNTs in cells were reduced by 55.2 and 72.4% after incubation with 5% ethanol and at 4 °C compared with the control, respectively. Besides, expression levels of endocytosis-related genes [END3 (Fig. 4c), END6 (Fig. 4d), Sla2 (Fig. 4e) and Rsp5 (Fig. 4f)] were increased after exposure to 0–376.4 mg/L O-SWCNTs suspensions. These data indicated that cellular uptake is predominantly an endocytosis process. Similar result was reported in other studies [27, 33].

Figure 3 clearly illustrated the penetration and internalization of O-SWCNTs into the cells. Direct penetration through the cell wall and membrane could be designated as one of the routes to deliver the O-SWCNTs into the cytoplasm. It was demonstrated that CNTs could readily permeate through the cell wall, driven by van der Waals forces or hydraulic forces [42]. Based on the combined results of Figs. 3 and 4, it can be concluded that internalization of O-SWCNTs in *S. cerevisiae* cells via: (1) direct penetration. O-SWCNTs were firstly adsorbed onto the cell wall surface, and then penetrated across the cell wall and membrane. After the penetration, O-SWCNTs distributed in the cytoplasm, vesicles, lysosomes and cell nucleus; (2) endocytosis. O-SWCNTs adsorbed and penetrated across the cell wall. Subsequently, the cell membrane was invaginated and wrapped up the O-SWCNTs. O-SWCNTs entered into the cells within the endosomes. The endosomes merged with lysosomes, and then lysosomes merged with vacuoles. The findings are somewhat similar to the results of previous studies [1, 47].

### Effects of O-SWCNTs on cellular structure

**Fig. 5:**
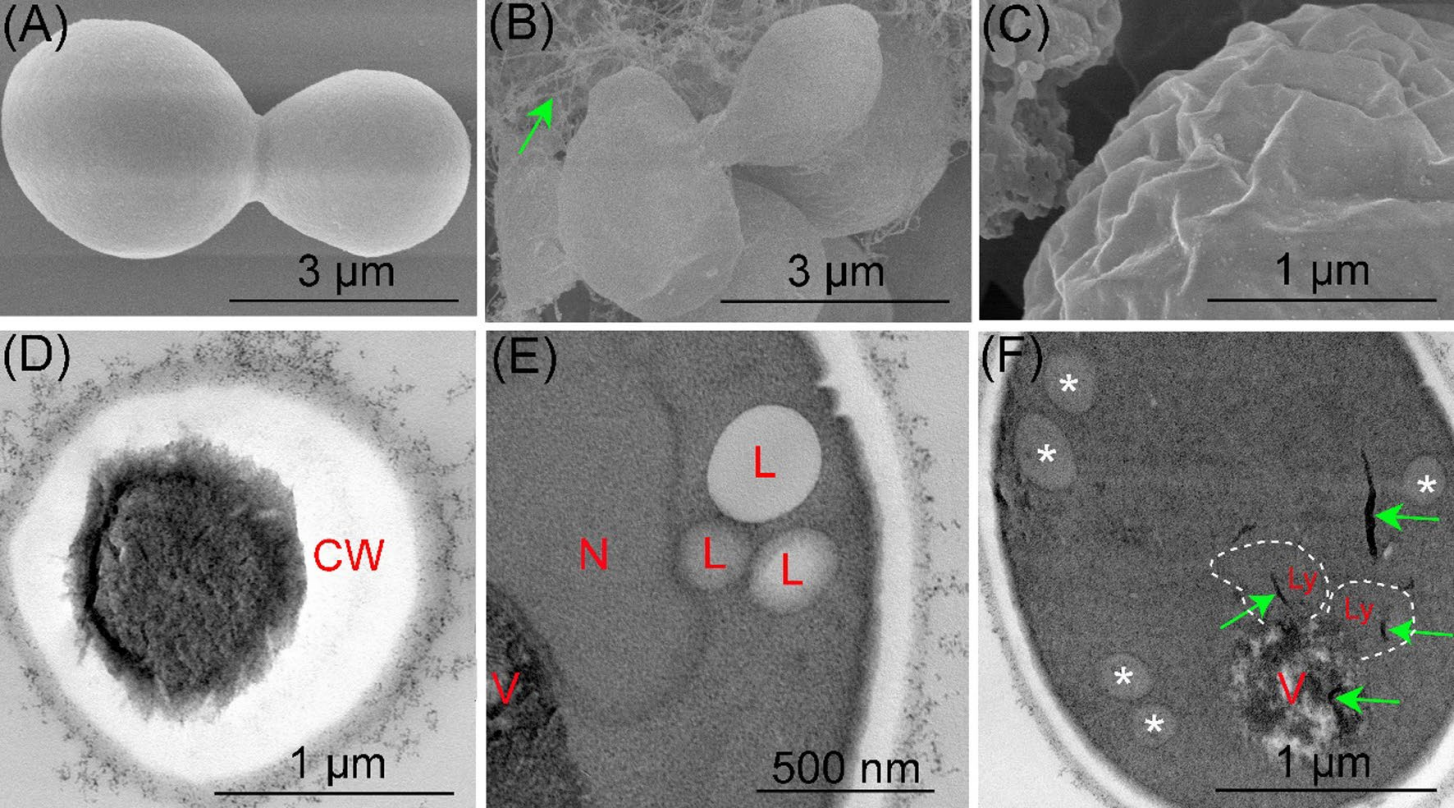
Effects of O-SWCNTs (green arrows) on cellular structure. **a** Surface of cells treated without O-SWCNTs was plump and smooth. **b**, **c** After exposure to O-SWCNTs, cells were deformed and shrank. **d** Cell wall of crinkled cell was incrassate, and its thickness was not uniform. Increased numbers of lipid droplets (**e**) and disruption of vacuoles (**f**) were also observed. *CW* cell wall, *V* vesicle, *N* nucleus, *L* lipid droplet, *Ly* lysosome, *mitochondrion

One of the CNTs-cytotoxicity mechanisms was proposed to be CNTs-induced cellular structure damages [22, 33]. As shown in Fig. 5a, surface of cells treated without O-SWCNTs was plump and smooth. After exposure to O-SWCNTs, cells were deformed and shrank (Fig. 5b, c). Cell wall of crinkled cell was incrassate, and its thickness was not uniform (Fig. 5d). Other damages, such as increased numbers of lipid droplets (Fig. 5e) and disruption of vacuoles (Fig. 5f) were also observed. Our findings are similar to the results reported by Bayat et al. [6], who investigated the effects of engineered nanoparticles on the cellular structure of *S. cerevisiae*. They demonstrated that increase in the number of lipid droplets, disruption or lack of vacuoles, shrinking of the cytosol and undulating appearance at the membrane were visible after exposure to CuO and Ag nanoparticle [6].

### ROS and antioxidant enzymes activities

**Fig. 6:**
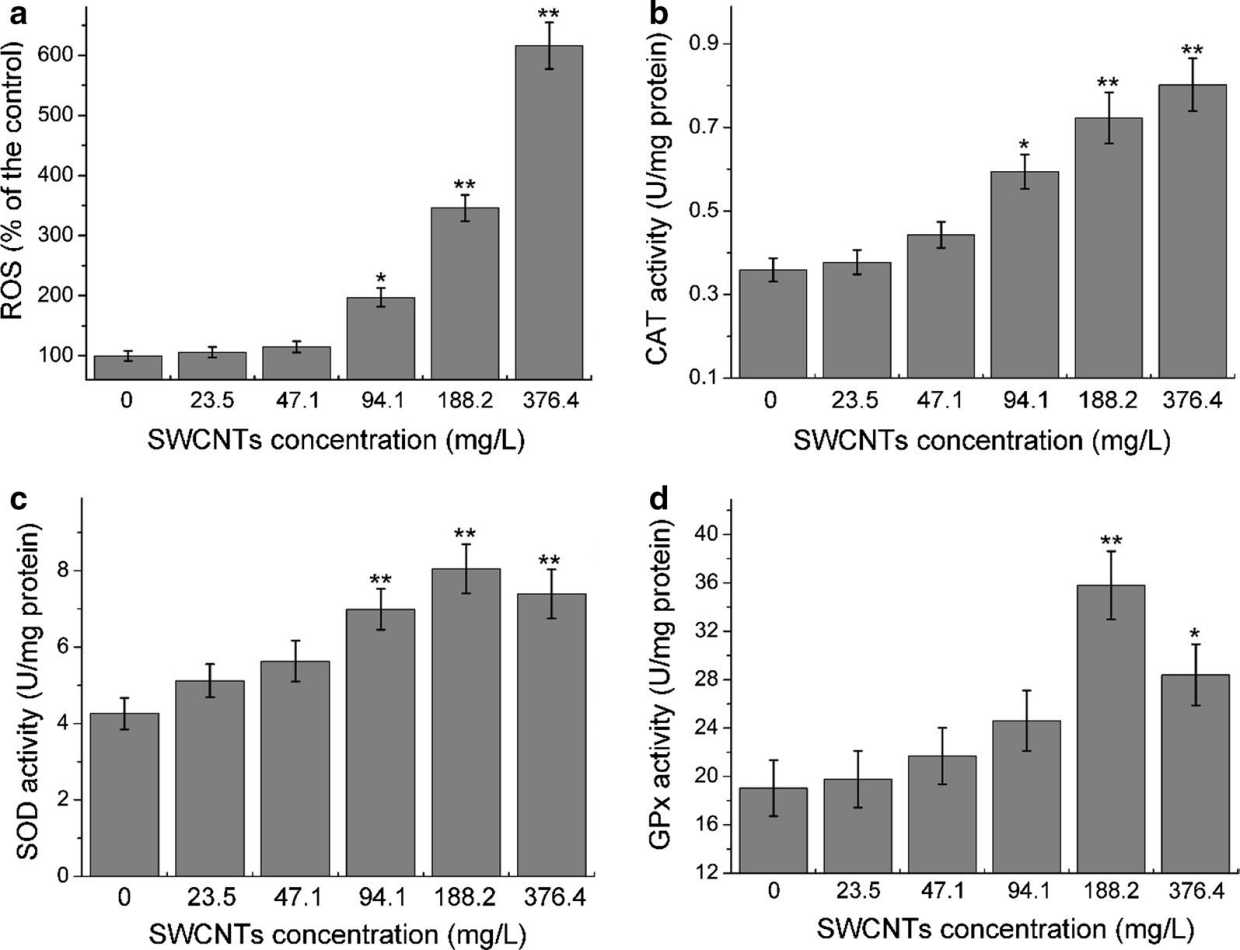
Effects of O-SWCNTs on ROS generation (**a**), CAT (**b**), SOD (**c**) and GPx (**d**) activities. Values are presented as mean ± SD. Values that are significantly different from the controls are indicated by asterisks (one-way ANOVA, *p < 0.05; **p < 0.01)

SWCNTs internalized into the cells would induce oxidative stress by producing ROS, which could undoubtedly be one of the factors responsible for the acceleration of cell apoptosis or death. Exposure to SWCNTs has been associated with altered levels of components in the antioxidant defense system, such as CAT, SOD and GPx [9, 25, 30]. Production of ROS and antioxidant enzymes activities were detected in the study. As shown in Fig. 6a, ROS was observably (*p *< 0.01) increased following exposure to 188.2 and 376.4 mg/L O-SWCNTs compared with the control. CAT activity showed a dose-dependent increase and dramatically increased (*p *< 0.01) at 188.2 and 376.4 mg/L (Fig. 6b). Interestingly, SOD (Fig. 6c) and GPx (Fig. 6d) activities were firstly increased and follow by decreases. Increases of CAT, SOD and GPx activities may be due to responses to the superoxide, high antioxidant enzymes activities can efficiently degrade superoxide. Decreases of SOD and GPx activities may be due to the depletion of SOD and GPx, or decrease in cell viability. Similar phenomenon was reported in other studies [32, 38]. In general, the data indicated that oxidative stress was induced following exposure to O-SWCNTs.

### Cell apoptosis

**Fig. 7:**
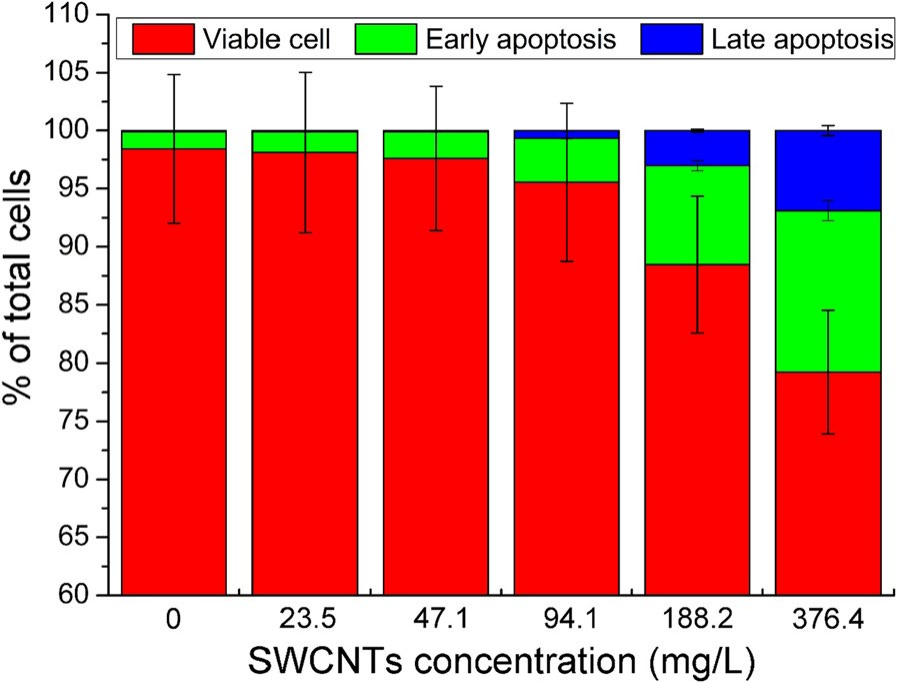
Flow cytometric analysis of apoptosis using Annexin V/PI staining. The annexin V-FITC^−^/PI^−^, annexin V-FITC^+^/PI^−^ and annexin V-FITC^+^/PI^+^ populations were regarded as viable cells, early apoptosis and late apoptosis, respectively. Values are presented as mean ± SD

Several studies reported that SWCNTs can induce cell apoptosis/necrosis. Besides, one of the most common SWCNTs mechanisms that lead to cytotoxicity is related to oxidative stress that can cause mitochondrial impairment and apoptosis [9, 36]. For example, Cheng et al. [9] investigated the effect of SWCNTs on rat aorta endothelial cells and demonstrated that SWCNTs induced apoptosis in the cells. Moreover, they also showed that ROS generation was involved in activation of the mitochondria-dependent apoptotic pathway [9]. To study whether O-SWCNTs can induce cellular apoptosis/necrosis, Annexin V/PI staining was performed and analyzed by flow cytometry. As shown in Fig. 7, there was no obvious apoptosis following exposure to 0–94.1 mg/L. However, it showed a significant increase (*p *< 0.01) after exposure to 188.2 (11.5%) and 376.4 (20.8%) mg/L for 24 h compared with the control. The data indicated that the increase of mortality was related to apoptosis.

### Mitochondrial transmembrane potential

**Fig. 8:**
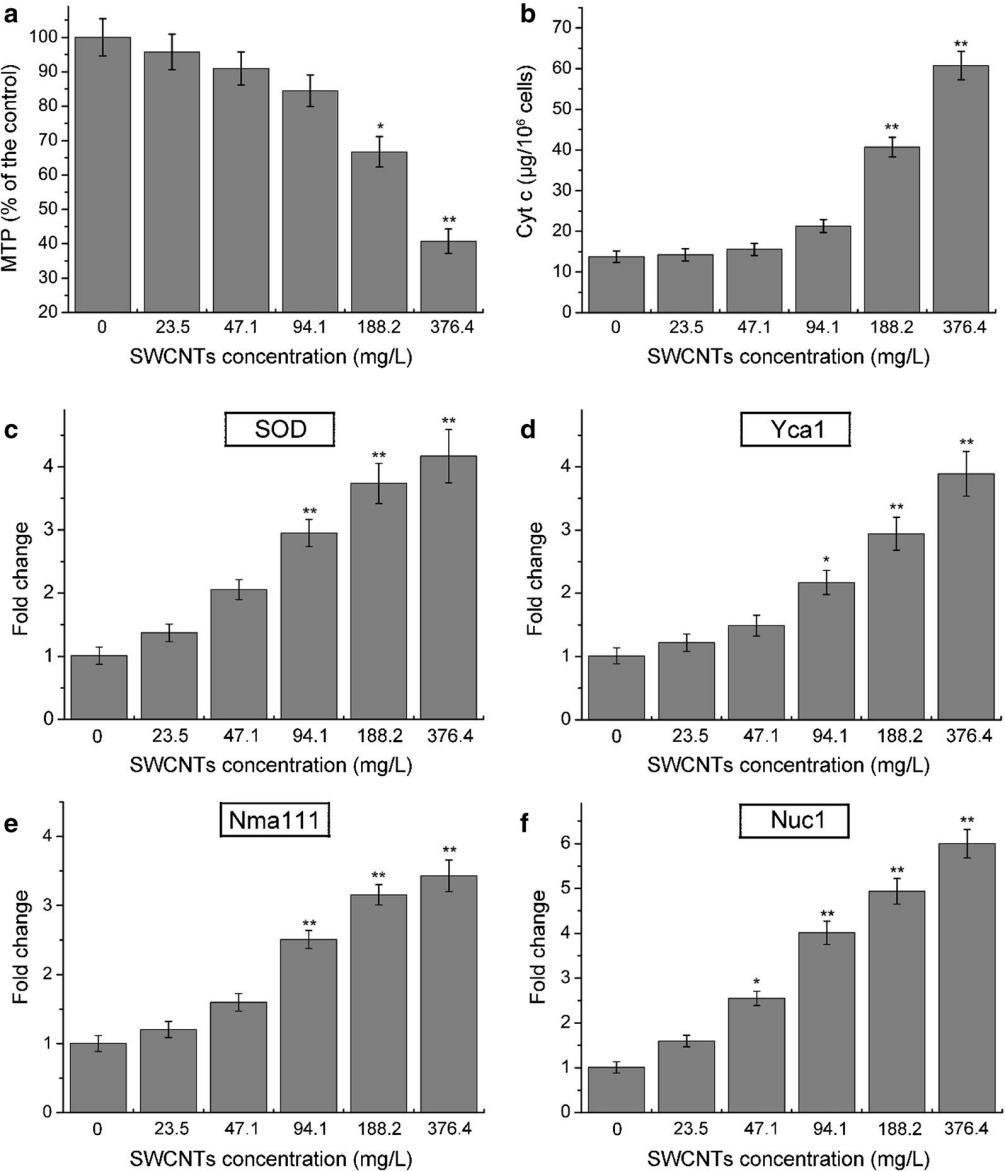
**a** Mitochondrial transmembrane potential (MTP) of *S. cerevisiae* cells was evaluated using JC-1, and measured by microplate reader. **b** Contents of cytochrome c in cytoplasm after exposure to 0–376.4 mg/L O-SWCNTs. Expressions of SOD (**c**), Yca1 (**d**), Nma111 (**e**) and Nuc1 (**f**) following exposure to O-SWCNTs (0–376.4 mg/L). Values are presented as mean ± SD. Values that are significantly different from the control are indicated by asterisks (one-way ANOVA, **p < 0.01)

Mitochondria have been suggested to play a key role in the apoptotic process. Their control of apoptosis has been described as: (1) maintenance of ATP production; (2) MTP and mitochondrial membrane permeability for the release of certain apoptogenic factors from the intermembrane space into the cytosol [24]. Reduction in MTP has been postulated to be an early and obligate event in the apoptotic signaling pathway [43]. In the study, changes of MTP after exposure to O-SWCNTs suspensions for 24 h were measured using JC-1 staining. As shown in Fig. 8a, MTP was significantly decreased (*p *< 0.01) at 376.4 mg/L compared with the control, indicating that the apoptosis induced by O-SWCNTs was related to mitochondrial impairment. The result is in accordance with previous studies [9, 36].

### Leakage of cytochrome c from mitochondria

Decrease of MTP is associated with the opening of the mitochondria permeability transition pore. Mitochondria become so permeable that they are unable to maintain a sufficient force to retain cytochrome c (Cyt c), thus, Cyt c will release from the mitochondria into the cytoplasm. Contents of Cyt c in cytoplasm were detected in the study. As shown in Fig. 8b, significant increases in Cyt c were observed compared with the control after exposure to 188.2 and 376.4 mg/L. The result indicated that mitochondrial membrane permeability was increased, and Cyt c was released from mitochondria to cytoplasm following exposure to O-SWCNTs.

### Expression of apoptosis-related genes

Superoxide dismutase gene encodes superoxide dismutase, which is a crucial enzyme in removing superoxide anion. It is sensitive to a variety of stress agents due to escalated oxidative stress. Moreover, it also plays a key role in the apoptotic process [21]. Yca1 mediates the apoptotic process, and encodes a yeast protein with structural homology and similar function to mammalian caspases [26]. Nma111 encodes a protein which belongs to the HtrA family of serine proteases, and its pro-apoptotic activity depends on its serine-protease activity [13]. Nuc1 has roles in mitochondrial recombination and apoptosis [5]. Expressions of SOD (Fig. 8c), Yca1 (Fig. 8d), Nma111 (Fig. 8e) and Nuc1 (Fig. 8f) were significantly increased following exposure to O-SWCNTs, indicating that *S. cerevisiae* cells were undergone apoptosis related to oxidative stress and mitochondrial impairment. The findings were consistent with previous studies [9, 30].

## Conclusion

Taken together, results so far indicated that: (1) O-SWCNTs (< 47.1 mg/L) show no obvious effects on *S. cerevisiae* cells; (2) O-SWCNTs can internalize in *S. cerevisiae* cells and distribute in cytoplasm, vesicles, lysosomes and cell nucleus; (3) internalization of O-SWCNTs in *S. cerevisiae* cells via direct penetration and endocytosis; (4) the maximum content of O-SWCNTs in *S. cerevisiae* cells is reached at 18 h after exposure; (5) O-SWCNTs (188.2 and 376.4 mg/L) induce apoptosis in *S. cerevisiae* cells, and oxidative stress is involved in activation of the mitochondria-dependent apoptotic pathway.

## Abbreviations

SEM: scanning electron microscope
TEM: transmission electron microscope
XPS: X-ray photoelectron spectroscopy
PBS: phosphate buffer solution
ROS: reactive oxygen species
SOD: superoxide dismutase
CAT: catalase
GPx: glutathione peroxidase
